# Effects of Heart Rate and Ventricular Wall Thickness on Non-invasive Mapping: An *in silico* Study

**DOI:** 10.3389/fphys.2019.00308

**Published:** 2019-04-05

**Authors:** Erick Andres Perez Alday, Dominic G. Whittaker, Alan P. Benson, Michael A. Colman

**Affiliations:** ^1^Knight Cardiovascular Institute, Oregon Health & Science University, Portland, OR, United States; ^2^School of Biomedical Science and Multidisciplinary Cardiovascular Research Centre, University of Leeds, Leeds, United Kingdom

**Keywords:** ECGi, non-invasive mapping, body surface potential, heart rate, cardiac hyperthrophy

## Abstract

**Background:** Non-invasive cardiac mapping—also known as Electrocardiographic imaging (ECGi)—is a novel, painless and relatively economic method to map the electrical activation and repolarization patterns of the heart, providing a valuable tool for early identification and diagnosis of conduction abnormalities and arrhythmias. Moreover, the ability to obtain information on cardiac electrical activity non-invasively using ECGi provides the potential for *a priori* information to guide invasive surgical procedures, improving success rates, and reducing procedure time.

Previous studies have shown the influence of clinical variables, such as heart rate, heart size, endocardial wall, and body composition on surface electrocardiogram (ECG) measurements. The influence of clinical variables on the ECG variability has provided information on cardiovascular control and its abnormalities in various pathologies. However, the effects of such clinical variables on the Body Surface Potential (BSP) and ECGi maps have yet to be systematically investigated.

**Methods:** In this study we investigated the effects of heart size, intracardiac thickness, and heart rate on BSP and ECGi maps using a previously-developed 3D electrophysiologically-detailed ventricles-torso model. The inverse solution was solved using the three different Tikhonov regularization methods.

**Results:** Through comparison of multiple measures of error/accuracy on the ECGi reconstructions, our results showed that using different heart geometries to solve the forward and inverse problems produced a larger estimated focal excitation location. An increase of ~2 mm in the Euclidean distance error was observed for an increase in the heart size. However, the estimation of the location of focal activity was still able to be obtained. Similarly, a Euclidean distance increase was observed when the order of regularization was reduced.

For the case of activation maps reconstructed at the same ectopic focus location but different heart rates, an increase in the errors and Euclidean distance was observed when the heart rate was increased.

**Conclusions:** Non-invasive cardiac mapping can still provide useful information about cardiac activation patterns for the cases when a different geometry is used for the inverse problem compared to the one used for the forward solution; rapid pacing rates can induce order-dependent errors in the accuracy of reconstruction.

## Introduction

Cardiovascular disease is a major contributor to reduced quality of life and mortality worldwide (Benjamin et al., 2017). Cardiac conditions such as heart failure, myocardial infarction, and hypertrophic/dilated cardiac myopathy are related to electrical dysfunction (i.e., arrhythmia) and typically result in reduced cardiac output. Diagnosis and treatment of these conditions presents a significant healthcare challenge, in part due to their dual electrophysiological-structural components. Short- and long-term adaptation of cardiac structure and ion channel expression, which includes reversible and irreversible remodeling associated with disease, further compounds the challenge. For example, cardiac hypertrophy, which is an important risk factor of heart failure and sudden cardiac death (Vriesendorp et al., 2015), is characterized by abnormal thickening of the heart muscle, usually resulting from increases in cardiac cell size, in order to compensate for inhibited contractile performance (Shimizu and Minamino, 2016). The particular manifestation of electrical dysfunction may therefore vary over the time-course of the condition; the ability to accurately map the electrical activity of the heart non-invasively over this whole period can offer significant advantages for the long-term management of such conditions.

Electrocardiographic imaging (ECGi) is a novel, painless and (relatively) economic method to map the electrical activation and repolarization patterns of the heart (Ghosh et al., 2011; Alday et al., 2016; Bear et al., 2016; Perez Alday et al., 2016; Zhang et al., 2016), and presents the possibility to better understand cardiac excitation patterns and provide *a priori* information to guide invasive surgical procedures, improving success rates and reducing procedure time (Silva et al., 2009; Dubois et al., 2015; Zhang et al., 2016). Based on solving the inverse problem of electrocardiography, with the heart acting as an electrical source inside the volume conductor of the body, ECGi aims to reconstruct the electrical activity on the surface of the heart using body surface potential (BSP) maps obtained from torso surface multi-array electrocardiogram (ECG) systems (Macfarlane et al., 2010; Rudy, 2013; Perez-Alday et al., 2017b). It depends on 3D heart and torso structures and therefore requires reconstructions of patients' cardiac and torso anatomy, which are typically acquired using the clinical imaging technologies of Magnetic Resonance Imaging (MRI) or Computed Tomography (CT). Due to the expense of these modalities, it may not be desirable to attain structural information from a patient repeatedly over the course of structural adaptions. However, the potential impact of using out-of-date structural information when performing ECGi is unclear.

In addition, previous studies have shown the influence of clinical variables, such as respiration (Langley et al., 2010; Baumert et al., 2013), body composition, (Zemzemi et al., 2015), and heart rate and body position (Appel et al., 1989; Goldenberg et al., 2006) on the ECG measurement. Based on these insights, adjusted ECG parameters (e.g., corrected QT interval) have improved the detection of patients at increased risk of cardiac arrhythmias (Kabir et al., 2016). It follows that such variables may also influence interpretation of BSP and ECGi data, but the nature of these relationships have yet to be systematically investigated.

The aim of this study was therefore to assess the effect of varying cardiac structure and electrical pacing rate on the accuracy of ECGi reconstructions. An *in silico* approach was used to provide clean and controllable data to compare reconstructions attained at multiple pacing rates and with underlying hypertrophic and dilated cardiac anatomy under sinus rhythm and ectopic focal excitation.

## Methods

The *in silico* approach utilized idealized, electrophysiologically heterogeneous human bi-ventricle models to simulate electrical excitation in control, dilated and hypertrophied conditions (sections “Virtual Bi-ventricle Models” to “Ventricular Simulation Protocols”). Ventricular activation was then combined with a heterogeneous torso model and the forward problem was solved to produce simulated BSP maps (section “Simulated Body Surface Potential”). The inverse solution, using multiple regularization approaches, was applied to the simulated BSP maps in order to produce ECGi epicardial potential reconstructions and compute activation patterns (section “Inverse Solution”). Multiple measures were used to quantify and compare results obtained under the different conditions (section “Analysis Methods”).

### Virtual Bi-Ventricle Models

**Figure 1:**
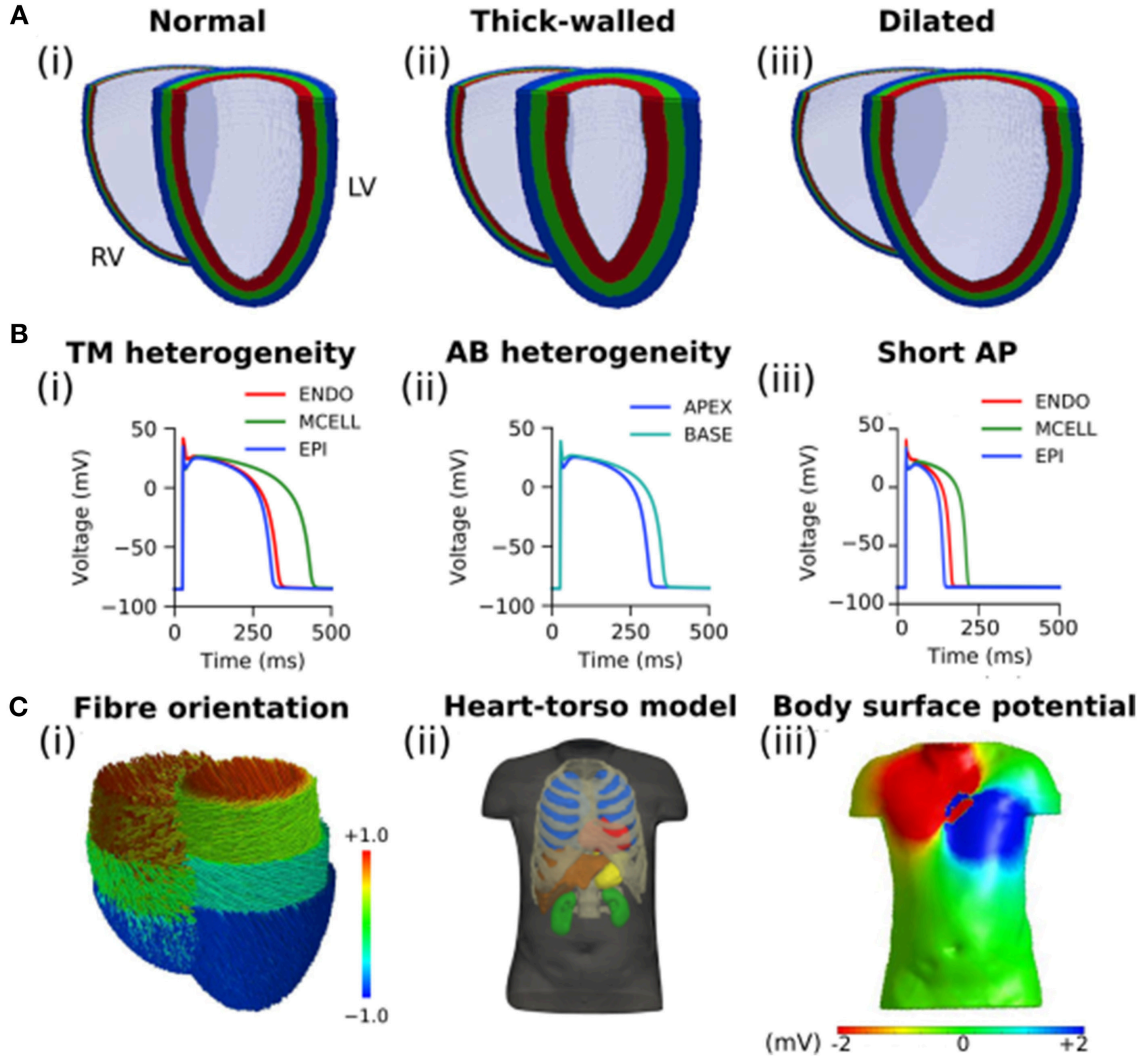
Computational models of the human ventricles and torso. **(A)** An open view of geometries representing (i) normal/control, (ii) thick-walled, and (iii) dilated human ventricles with the epicardial (blue), mid-myocardial (green), and endocardial (red) segmented regions shown. **(B)** Single cell ventricular action potentials representing (i) transmural (TM) heterogeneity in cells from the endocardium (ENDO), mid-myocardium (MCELL), and epicardium (EPI), (ii) apico-basal (AB) heterogeneity (shown for EPI cells), and (iii) short single cell action potentials used in this study. All models are uncoupled single cells paced at a cycle length of 1,000 ms. **(C)** (i) Fiber orientation (normalized z component of primary fiber; red and blue indicate parallel to the long axis of the heart from apex to base, green indicates perpendicular to the long axis of the heart); (ii) Heart-torso model used to compute the (iii) body surface potential.

Idealized human bi-ventricle geometries were constructed as structured finite difference grids, wherein the left and right ventricles (LV and RV, respectively) were modeled as thick- and thin-walled truncated ellipsoids, respectively. A control (normal) geometry was constructed in order to have physiologically-accurate ventricular wall thicknesses (12–15 and 3–5 mm for LV and RV, respectively; Ho and Nihoyannopoulos, 2006; Ho, 2009) and volumes (~150–210 mL for the LV in human males; Alfakih et al., 2003; Clay et al., 2006), and the overall size and ventricular curvature were qualitatively matched against multiple existing human ventricle datasets (Seemann et al., 2006; Benson et al., 2011; Keller et al., 2011). From this, two more geometries were created by increasing either the wall thickness or the short axis diameter by 50%. In total three cases were considered: (i) normal, (ii) thick-walled (hypertrophied), and (iii) dilated ventricles (Figure 1A). A spatial resolution of Δ*x* = Δ*y* = Δ*z* = 0.5 mm was used, which gave ~2 × 10^6^ nodes in tissue, to facilitate high-throughput generation of multiple datasets for BSP and ECGi analysis. Measurements of the LV volume, LV wall thickness, and RV wall thickness from the developed geometries are given in Table 1.

**Table 1:** A summary of dimensions in developed idealized ventricular geometries.

	**Control**	**Thick-walled**	**Dilated**
LV wall volume (mL)	196.56	249.77	255.29
LV wall thickness (mm)	12.00	18.00	12.00
RV wall thickness (mm)	4.00	6.00	4.00

In each case, a simple ruled-based model was implemented to assign myocardial fiber orientations (Figure 1Ci) using a standard approach based on rules proposed by Streeter et al. (1969). A value of the helix angle, α, was assigned to each node of the grid, given by

(1) $$\begin{array}{r} \alpha =R{\left(1-2d\right)}^{n}, \end{array}$$

where *R* is the transmural rotation (varying from +*R* at the endocardium to –*R* at the epicardium), *d* is the normalized transmural depth (varying from 0 at the endocardium to 1 at the epicardium), and *n* determines the transmural variation in helix angle (e.g., *n* = 1 is linear, *n* = 3 is cubic). For all simulations in this study, *R* was set to 60°, giving a transmural rotation in helix angle of 120°, similar to that observed in existing human ventricular datasets (Seemann et al., 2006; Benson et al., 2010), and *n* was set to 1 (Benson et al., 2008). The transverse angle was assumed to be 0° as it has been shown to be constantly around 0° throughout the ventricles (Seemann et al., 2006), and no sheetlet structure was incorporated, as this has been suggested to show great variability between hearts (Benson et al., 2008). A small degree of smoothing was applied where the right ventricle joins the ventricular septum, to ensure a smooth transition in helix angles.

### Single Cell Model of Human Ventricles

To simulate the action potential (AP) of human ventricular myocytes, the 2006 version of the Ten Tusscher et al. model was used (Ten Tusscher and Panfilov, 2006), which accounts for distinct electrophysiological differences in cells from the ventricular endocardium (ENDO), mid-myocardium (MCELL), and epicardium (EPI; Figure 1Bi). The bi-ventricle models were segmented into 40% ENDO, 30% MCELL, and 30% EPI cells (Figure 1A), similar to previously used ratios (Adeniran et al., 2011, 2017). The existing transmural heterogeneity was increased by adjusting the ENDO:EPI:MCELL ratio of rapid delayed rectifier potassium current, *I*_Kr_, maximal conductance to 1.0:1.6:1.0 (Adeniran et al., 2011; Whittaker et al., 2017). This was based on transmural measurements of hERG mRNA expression (Szabó et al., 2005), and was necessary to reproduce the longer AP of ENDO compared to EPI cells (Glukhov et al., 2010; Boukens et al., 2015). Furthermore, a linear gradient in the conductance of transient outward potassium current, *I*_to_, and slow delayed rectifier potassium current, *I*_Ks_, was introduced along the apex-base (AB) axis (Keller et al., 2011; Alday et al., 2016). Briefly, maximal conductance of *I*_to_ and *I*_Ks_ were reduced by a maximum of 50% in basal cells relative to apical cells in order to reproduce apico-basal heterogeneity (Figure 1Bii), giving a roughly 50 ms longer AP duration in basal cells than in apical cells (Szentadrassy et al., 2005). The maximal conductance of current *x* from cell type *y, g*_*x, y*_, was given by

(2) $$\begin{array}{r} g_{x,y}=g_{Base,y}+\left(g_{Apex,y}-g_{Base,y}\right)\cdot f_{AB}, \end{array}$$

(3) $$\begin{array}{r} f_{AB}=\frac{z-z_{Base}}{z_{Apex}-z_{Base}}, \end{array}$$

where *g*_Apex,y_ and *g*_Base,y_ are maximal values of the conductance of cell type *y* at the apex and base, respectively, *f*_AB_ is a gradient factor which depends linearly on the value of the *z* co-ordinate which lies along the AB axis (varying from 1 at the apex to 0 at the base), and *z*_Base_ and *z*_Apex_ are the values of the *z* coordinate at the apex and base, respectively. No electrophysiological differences were incorporated between the LV and RV (Keller et al., 2011).

### Modeling Action Potential Propagation

The monodomain equation was used to describe the propagation of APs in the bi-ventricle geometries:

(4) $$\frac{\partial V}{\partial t}=\nabla \left(D\nabla V\right)-\frac{I_{\text{ion}}}{C_{\text{m}}},$$

where *V* is the transmembrane voltage, **D** is the global conductivity tensor, *I*_ion_ is the total ionic current, and *C*_m_ is the membrane capacitance. Equation (4) was solved numerically using a finite-difference PDE solver based on the explicit forward Euler method, using an operator splitting technique and an adaptive time step with minimum and maximum time steps of Δ*t*_*min*_ = 0.02 ms and Δ*t*_*max*_ = 0.2 ms, respectively (Benson et al., 2010). As axially-symmetric anisotropy was assumed, two principal values of the diffusion coefficient were required: *D*_||_, the longitudinal value of the conductivity which describes propagation in the fiber direction, and *D*_⊥_, the transverse value, which describes propagation orthogonal to fibers. The diffusion tensor can thus be written as

(5) $$\text{D}=D_{⊥}\text{I}+\left(D_{∥}-D_{⊥}\right)\text{A}A^{\text{T}},$$

where **I** is the identity matrix, **A** is a unit vector giving the fiber direction, and **A**^T^ is the transpose of **A**.

The longitudinal value of the conductivity, *D*_||_, was set to 0.18 mm^2^ms^−1^ in this study, which gave a conduction velocity of 70 cms^−1^ in the fiber direction (Benson et al., 2007), in agreement with experimental measurements of conduction velocity along fibers in human ventricular tissue (Taggart et al., 2000). An anisotropic conductivity ratio of *D*_||_:*D*_⊥_ = 4:1 was used (Benson et al., 2007; Whittaker et al., 2017).

### Ventricular Simulation Protocols

Sinus rhythm activation of the ventricles was elicited by stimulating a series of 28 localized patches (with diameters of ~9–12 mm) in quick succession along the endocardial wall (stimulus amplitude and duration −52 pA/pF and 1 ms, respectively, where the wavefront was initiated in the intra-ventricular septum before spreading from apex to base throughout the left and right ventricles. This gave a total activation time of ~65 ms in the control geometry, in good agreement with the classic results of Durrer et al. (1970). For studying the effects of ectopic activity in the ventricles, four prescribed locations which could be easily identified in each of the geometries were chosen as “ectopic stimulus” sites: (i) the right ventricular lateral wall (RV-LAT), (ii) the intra-ventricular septum (SEP), (iii) the left ventricular lateral wall (LV-LAT), and (iv) the left ventricular apex (LV-Apex). In each case, localized −52 pA/pF stimuli of 1 ms duration were applied over 5 mm.

### Simulated Body Surface Potential

The ventricular model was placed into a previously developed biophysically-detailed computational three-dimensional heart-torso model which accounts for the distinct structures of the lungs, liver, blood masses, stomach, spleen, kidneys, ribs and spinal cord, and the respective electrical conductivities (Perez-Alday et al., 2015) (Figure 1Cii). This model has been previously used to develop an algorithm to diagnose atrial ectopic origin from multi lead ECG systems and ventricular ischemia (Alday et al., 2016; Perez-Alday et al., 2017a). Details of the torso model development, validation and simulation protocols can be found in Perez-Alday et al. (2015). Briefly, the heart-torso algorithm previously developed was used to solve the forward problem and obtain BSP maps (Figure 1Ciii) in each of the cases. The potential on the surface of the body was obtained from the 3D ventricular model using Salu's approach (Salu, 1980), utilizing the Boundary Element Method and Green's identities to solve the Poisson equation (Macfarlane et al., 2010).

### Inverse Solution

An inverse solution was developed, extending previously published preliminary work (Alday et al., 2016). Briefly, and based on prior work from Ramanathan and Rudy (2001), surface to surface torso-heart matrix was calculated using Barr's approach, where an equivalent potential distribution on a closed surface is used to build the homogenous heart-torso matrix (Barr et al., 1977); note therefore that, whereas the forward problem is solved on a heterogeneous torso, the inverse solution is provided on a homogeneous torso model. From the BSP maps, a previously developed inverse problem algorithm using Tikhonov regularization using Generalized Single Value Decomposition (GSVD) numerical approach was used to obtain the activation on the surface of the heart (Hansen, 1998). The potentials on the surface, *x*, were obtained by solving Equation (6):

(6) $$x=\underset{x}{\min}\left\{∥Zx-y{∥}_{2}+\lambda^{2}∥Rx∥\right\},$$

where *Z* is the transfer matrix, *y* represents the BSP vector, λ is the regularization parameter obtained using the L-curve (Hansen, 1992), and *R* is the regularization operator. Zero (Identity matrix, *R* = I), First (Gradient operator, *R* = ∇), and Second (Laplace operator, *R* = Δ) order Tikhonov were used to regularize the solution. The GSVD technique was used to solve Equation (6) in each case.

As an ill-posed problem, noisy signals can have an important effect on the reconstructed maps. Whereas it is common in modeling studies of ECGi to include additional white noise, this was not performed in this study for the bulk of our analysis. Please see “Discussion: Limitations” for further details on the inclusion of noise and its impact.

### Analysis Methods

Epicardial potentials were reconstructed from the BSP obtained at each instant of time for each geometry and activation case. Activation maps were calculated by computing maximal negative slope at each node at each time step (Gage et al., 2017). An example of original and reconstructed epicardial potential snapshots and the corresponding activation maps is shown in the Section S1 in Supplementary Material. To quantify the differences between the BSP and reconstructed activation maps for each of the geometry cases, three difference methods were used (Bear et al., 2015, 2018b):

1. Voltage root mean squared (RMS):
  $$\begin{array}{l} RMS=\;\sqrt{\frac{\sum_{i=1}^{N}\emptyset_{i}^{2}}{N}}; \end{array}$$
2. Relative RMS error (rRMSe):
  $$rRMSe=\sqrt{\frac{\sum_{i=1}^{N}{\left({\emptyset_{i}}^{\prime }-\emptyset_{i}\right)}^{2}}{\sum_{i=1}^{N}{\left({\emptyset_{i}}^{\prime }\right)}^{2}}};$$
3. Pearson correlation coefficient (PCC):
  $$PCC=\frac{\sum_{i=1}^{N}\left({\emptyset_{i}}^{\prime }-\emptyset_{i}\right)\left({\emptyset_{i}}^{\prime }-\emptyset_{i}\right)}{\sqrt{\sum_{i=1}^{N}{\left({\emptyset_{i}}^{\prime }-\emptyset_{i}\right)}^{2}{\left({\emptyset_{i}}^{\prime }-\emptyset_{i}\right)}^{2}}},$$

where *N* is the number of elements in the mesh (torso or epicardial elements), ∅ is the potential reconstructed or measured and ∅′ is the original simulated potential, while $\bar{\emptyset }$ and ${\bar{\emptyset }}^{\prime }$ are the mean potential values across all elements of the mesh. RMS gives an estimation of the variability of the signal. rRMSe gives an estimation of the variability between two methods. PCC is the measure of the correlation between two variables. The analysis was performed at each temporal snapshot of the ventricular activation.

To investigate the focus location accuracy of the inverse solutions, the Euclidean distance (ED) was calculated at the center of the earliest activation: $||ED||=\sqrt{{\left(r^{\prime }-r\right)}^{2}}$, where *r* is the center of activation of the reconstructed potential in the 3D Euclidean space and *r*′ is the center of activation of the original simulated data. The Euclidean distance was calculated for all the ectopic cases and a median value is reported in this study.

### Investigating the Effect of Using the Incorrect Geometry for ECGi

The impact of using only an initial patient anatomical reconstruction when performing ECGi, which doesn't capture any structural remodeling which may have occurred between the time of the scan and any present measurements, was investigated: Ectopic ventricular activation was simulated on all three geometries (control, thick-walled, and dilated; section “Virtual Bi-ventricle Models”) and used to solve the forward problem and produce BSP maps; the ECGi reconstruction was performed using only the control geometry, representing the initial patient scan. Quantification of errors and correlations were performed by comparing the reconstruction with the control geometry activation for each matched ectopic location, such that geometrical differences don't have to be accounted for.

### Investigating the Effect of Heart Rate on ECGi

The effect of heart rate on the epicardial reconstructions obtained using the three Tikhonov regularization methods was assessed for focal excitations using the control geometry paced at basic cycle lengths (BCL) of 1,000, 750, 500, 300, and 150 ms [corresponding to pacing rates of 60, 80, 120, 200, and 400 Beats per Minute (BPM), respectively]. Shortening of the AP (Figure 1Biii) induced by a five-fold increase in the conductance of I_Kr_ and I_Ks_ was employed to sustain the most rapid excitation rate (BCL = 150 ms). All other data were produced using the control AP models.

## Results

First, the impact of the different geometries (control, thick-walled, and dilated) on simulated BSP under control pacing conditions were compared to illustrate recapitulation of activation pattern and ECG differences observed under these conditions in the *in silico* framework (section “Simulated Body Surface Potential Under Different Conditions”). Then, the potential errors induced by using out-of-date and inaccurate cardiac anatomical reconstructions when performing ECGi was assessed (section “Effects of Wall Thickness and Heart Size on Non-invasive Cardiac Maps”). Finally, we investigated the effect of heart rate on the accuracy of reconstructed activation patterns using the different regularization approaches (section “Effects of Heart Rate on Non-invasive Cardiac Maps”).

### Simulated Body Surface Potential Under Different Conditions

**Figure 2:**
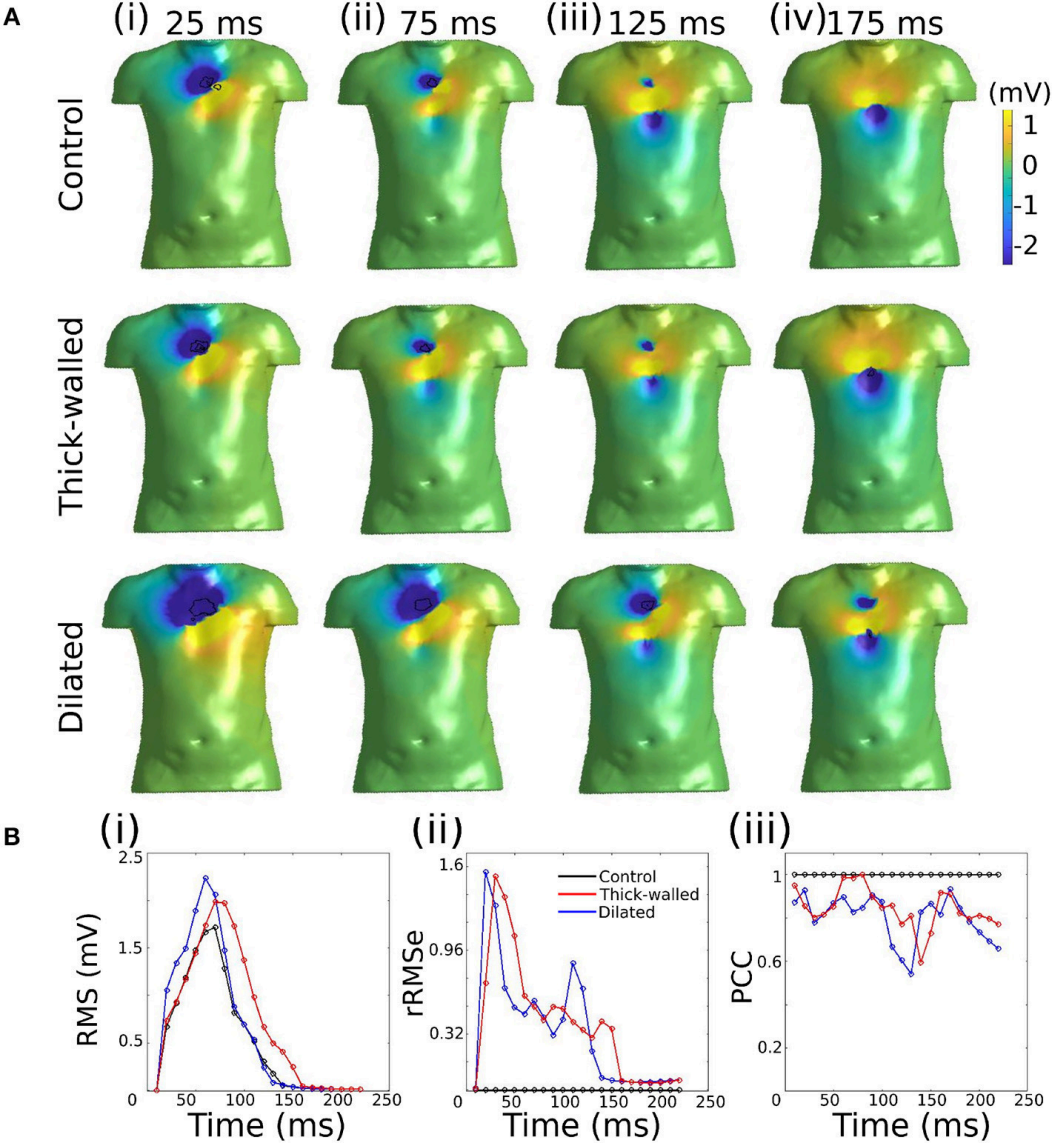
Comparison of simulated BSP obtained from the three different geometries during ventricular activation. **(A)** BSP obtained for control case, the thick-walled and dilated geometry during simulated ectopic activation initiated in the right ventricular lateral wall (RV-LAT) at different instants of time: (i) 25 ms, (ii) 75 ms, (iii) 125 ms, and (iv) 175 ms. **(B)** (i) RMS for each case and (ii) rRMSe and (iii) PCC calculated vs. the control case.

The effects of the different geometries on the BSP were quantified by comparing the thick-walled and dilated geometries vs. the control during ventricular activation (Figure 2). Small differences were observed in the BSP maps at different instants of time (Figure 2A), quantitative measurements are plotted for comparison. A similar RMS was obtained for the three cases which produced relatively small rRMSe values (Figure 2B). However, the largest values were observed early during the activation sequence (first 75 ms). A good agreement between the signal was observed for both cases (average PCC > 0.8), however, at mid activation time (between 125 and 175 ms) the values dropped significantly, with the dilated condition resulting in the smallest correlation.

### Effects of Wall Thickness and Heart Size on Non-invasive Cardiac Maps

**Figure 3:**
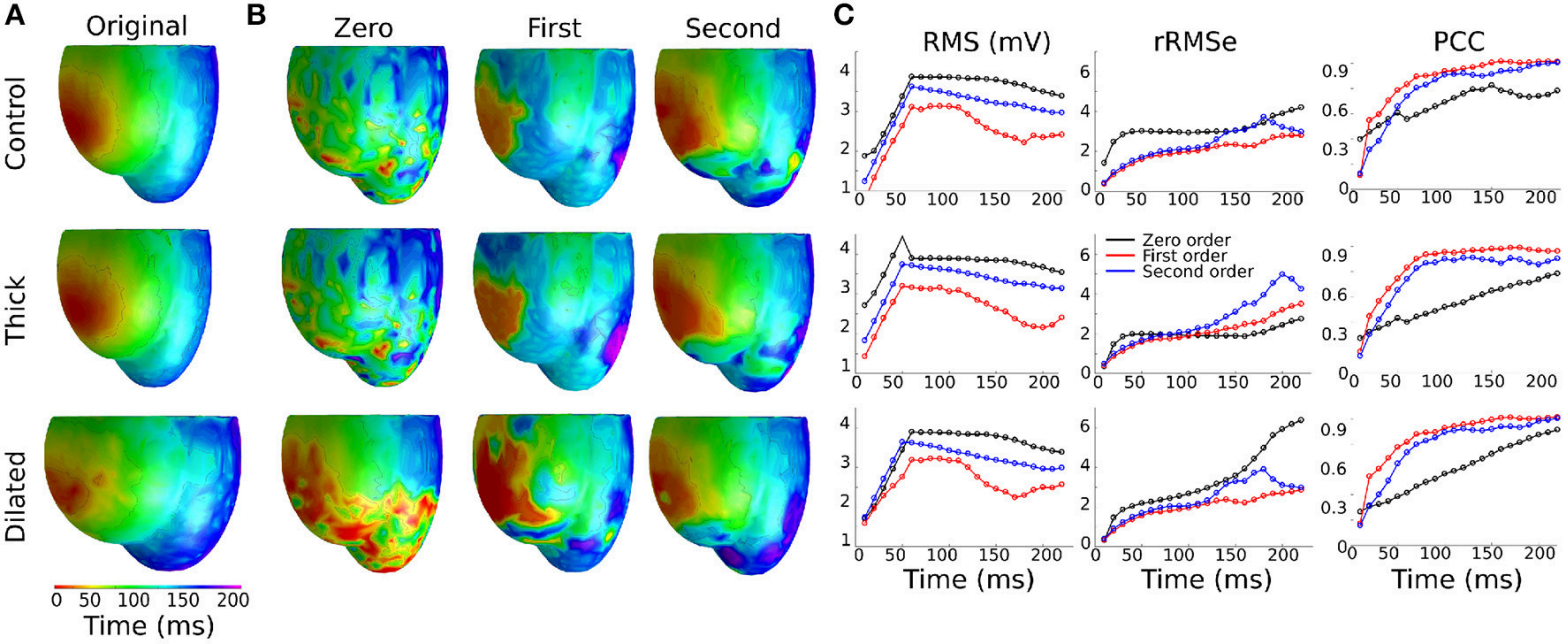
Effects of heart geometry on the reconstructed epicardial potentials. **(A)** Original simulated ectopic activation initiated in the right ventricular lateral wall (RV-LAT) on all three geometries. **(B)** Reconstructed activation maps using Zero, First and Second order Tikhonov regularization using different geometries for the forward problem but the control/normal geometry for the inverse problem. Activation patterns were computed as the time of maximum negative first derivative d*V*/*d*t at each location. **(C)** RMS, rRMSe, and PCC calculated to quantify the differences between the reconstructed activation patterns using Zero (black), First (blue) and Second (red) order Tikhonov regularization.

Data are illustrated for a single ectopic site only (RV-LAT—Figure 3) and summarized for all sites (Table 2). During the initial excitation phase (75 ms), similar small RMS and rRMSe values were observed for the three cases (Figure 3). During the mid and later activation times, RMS and rRMSe values were more dependent on the order of the regularization than the geometry, with First and Second order giving the smallest errors. Similarly, PCC values were considerably larger using First and Second order compared to the Zero order, and the Zero order displayed the most unique and geometry-dependent temporal evolution. The general increase in correlation over the time of the activation sequence is attributed to the increase in area of active tissue. RMS, rRMSe, and PCC were similar for all three heart geometries, although in general the control geometry exhibited the smallest errors and largest correlation and the dilated geometry exhibited the largest errors and smallest correlation (Figure 3; Table 2).

**Table 2:** A summary of the effects of geometry on RMS, rRMSe, and PCC of reconstructed activation maps using the three different regularization methods.

**Tikhonov order**	**Metrics**	**Control – mean** **(SD)**	**Dilated – mean** **(SD)**	**Thick – mean** **(SD)**
Zero order	RMS	3.45 (0.60)	3.74 (0.43)	3.65 (0.62)
	rRMSe	3.09 (0.57)	3.94 (0.45)	3.33 (0.63)
	PCC	0.673 (0.108)	0.570 (0.152)	0.603 (0.120)
First order	RMS	3.03 (0.59)	3.27 (0.46)	3.12 (0.45)
	rRMSe	2.34 (0.87)	2.77 (0.78)	2.98 (0.64)
	PCC	0.794 (0.210)	0.754 (0.213)	0.773 (0.120)
Second order	RMS	2.48 (0.59)	2.68 (0.48)	2.63 (0.44)
	rRMSe	1.97 (0.64)	2.65 (1.26)	2.39 (0.93)
	PCC	0.869 (0.106)	0.769 (0.121)	0.778 (0.140)

**Figure 4:**
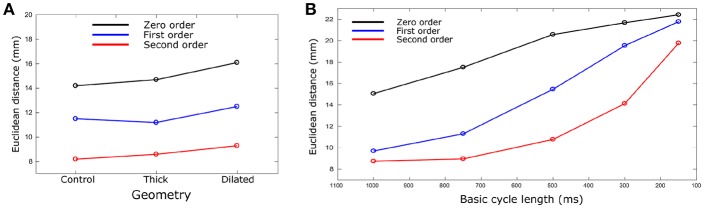
Euclidean distance vs. **(A)** geometry and **(B)** BCL. Euclidean distances were calculated for each geometry case and BCL for Zero (black), First (Blue), and Second (red) order Tikhonov regularization. For **(B)**, only control geometry was used. Data are the mean for all ectopic sites.

The calculated ED, measuring the error in correlation between real and identified focus location, varied for each geometry using the three Tikhonov methods (Figure 4A). Smaller values were observed for the Second order method (compared to Zero and First) and the control geometry (compared with thick and dilated, with dilated giving the largest values). However, the differences observed between geometries was less significant than that between methods.

### Effects of Heart Rate on Non-invasive Cardiac Maps

The ED was calculated and compared for each different heart rate and Tikhonov method (Figure 4B). The Second order method in all the cases produced the smaller ED values. A marked increase in the ED was observed when the heart rate was increased for all methods, which also resulted in convergence of the solutions obtained using the different methods at the most rapid rate.

**Figure 5:**
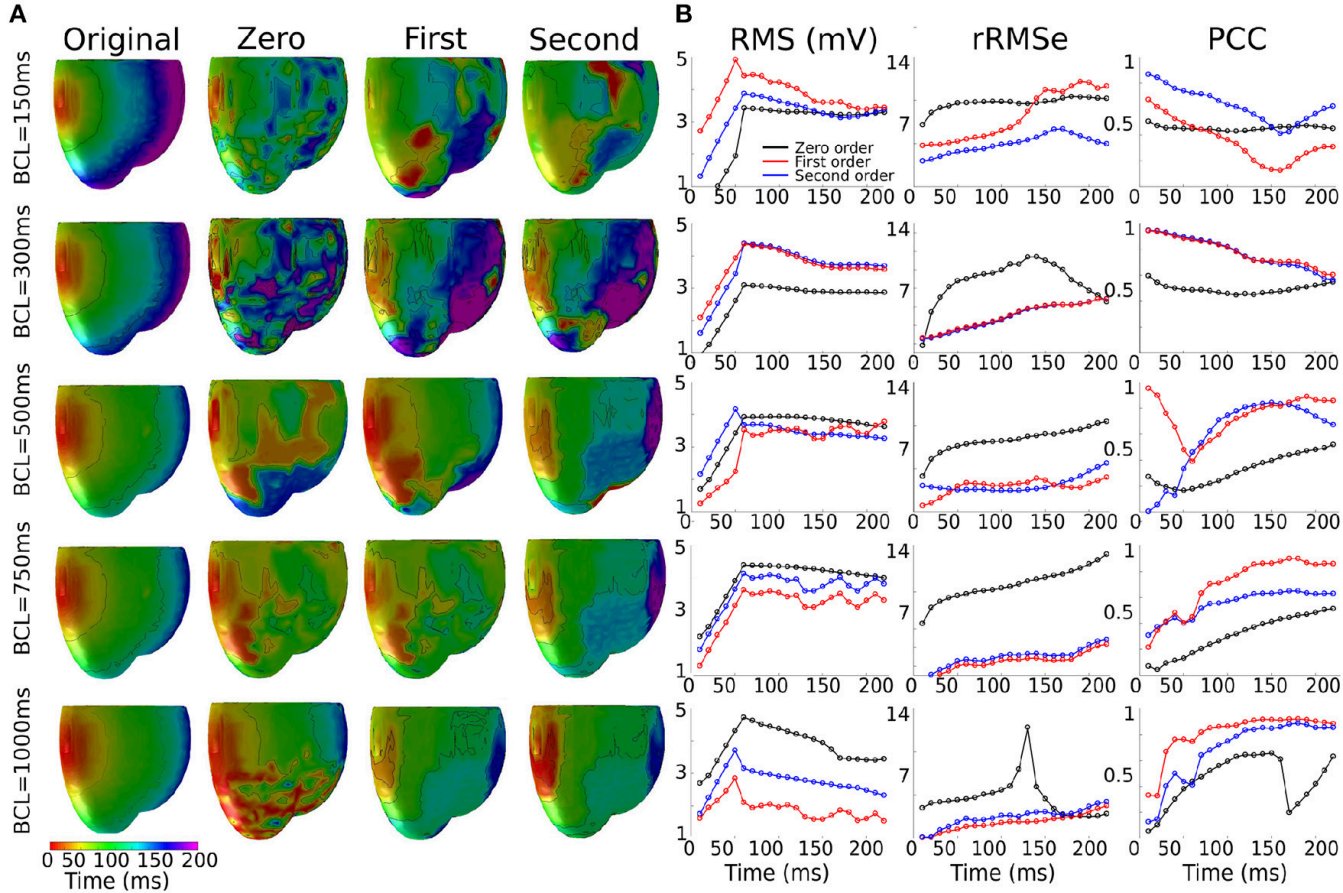
Effects of the heart rate on the reconstructed activation maps. **(A)** 3D activation maps at different basic cycle lengths (BCLs): 150, 300, 500, 750, and 1,000 ms; activation time is given as the maximum negative slope of each local membrane potential. **(B)** RMS, rMSe and PCC measure calculated using Zero (black), First (blue), and Second (red) order Tikhonov regularization of an ectopic activation starting on the middle of the left ventricle (LV-LAT).

The reconstructed activation patterns were compared across the different pacing rates; illustrative data for the LV-LAT site are shown in Figure 5 and data from all ectopic sites are summarized in Table 3. At the slowest pacing rates (BCL = 1,000 and 750 ms), corresponding to normal heart rates in healthy patients (60 and 80 BPM, respectively), the Zero order method resulted in the larger rRMSe values and lower PCC values and contained the most noise. Both the First and Second order methods resulted in lower rRMSe values and larger PCC values over the temporal range of excitation (Figure 5—BCL = 1,000 and 750 ms), with the Second order in general performing the best, in congruence with the ED values (Figure 4B). The PCC for all methods in general increased over the time of the activation. At the most rapid rates (Figure 5—BCL = 300 and 150 ms), the temporal evolution of the PCC for First and Second order reversed, decreasing over the activation time, whereas the Zero order remained largely flat. The initial larger correlation for First and Second order compared to the slow pacing rates did not correspond to small ED and therefore was not a result of accurate reconstruction of the initial phase of excitation. The differences between the methods decreased at these rapid rates, largely due to an increase in the errors associated with First and Second order with no corresponding change to the Zero order solution (Figure 5, BCL = 150 and 300 ms). In all conditions, the Zero order approximation presented the most noise, but the reconstruction at the rapid excitation rates was more stable and comparable with the Second and First order (Table 3).

**Table 3:** A summary of the effects of heart rate on reconstructed activation maps using the three different regularization methods.

**Tikhonov order**	**Metrics**	**BCL 1,000 ms**	**BCL 750 ms**	**BCL 500 ms**	**BCL 300 ms**	**BCL 150 ms**
Zero order	RMS	3.85 (0.55)	3.96 (0.62)	3.53 (0.65)	3.67 (0.59)	3.78 (1.02)
	rRMSe	7.64 (2.3)	7.81 (1.75)	8.31 (1.41)	8.77 (2.23)	8.70 (0.71)
	PCC	0.646 (0.190)	0.628 (.154)	0.626 (0.119)	0.629 (0.103)	0.623 (0.107)
First order	RMS	2.74 (0.39)	3.61 (0.61)	4.16 (0.66)	4.59 (0.63)	4.20 (0.61)
	rRMSe	2.82 (0.96)	2.94 (1.01)	3.14 (0.93)	4.91 (1.46)	4.92 (1.06)
	PCC	0.820 (0.169)	0.789 (0.106)	0.757 (0.280)	0.713 (0.122)	0.686 (0.130)
Second order	RMS	1.89 (0.31)	2.11 (0.61)	2.20 (0.62)	2.68 (0.55)	2.82 (0.51)
	rRMSe	2.43 (1.06)	2.44 (1.01)	2.92 (0.83)	3.98 (1.40)	4.17 (1.89)
	PCC	0.862 (0.138)	0.839 (0.213)	0.813 (0.148)	0.789 (0.107)	0.705 (0.160)

## Discussion

### Summary

In this study, we used an *in silico* approach to evaluate the impact of different ventricular anatomical morphologies and heart rate on the accuracy of epicardial reconstructions attained through the application of the inverse solution to the BSP. We have demonstrated that the different cardiac anatomical states resulted in small but measurable differences in the BSP (Figure 2). Furthermore, we demonstrated that differences between actual underlying cardiac anatomy (i.e., the heart model on which electrical activation was simulated) and the reconstructed anatomy (i.e., the heart model on which the inverse solution was applied) led to errors in the reconstruction of both epicardial potential maps and activation patterns (Figure 3; Table 2). However, the location of the ectopic focal excitation was still largely correctly estimated, even with the incorrect geometry used for reconstruction (Figure 4). Moreover, we have demonstrated an important heart rate dependency of the correlation coefficients and reconstruction errors (Figure 5; Table 3). In general, the Second order regularization approach produced the smallest errors and largest correlation.

### Clinical Importance

ECGi is a powerful and rapidly developing approach to non-invasively map patients' cardiac electrical activity in the clinic. The method aims to overcome some of the numerous challenges related to effective non-invasive characterization of human anatomy and electrophysiology. Previous studies have shown the usefulness of this non-invasive method to provide information to guide ablation procedures (Dubois et al., 2015; Rodrigo et al., 2017) and identify potential patients for whom cardiac resynchronization therapy would be successful (Silva et al., 2009; Rudy, 2013; Bear et al., 2018a). In addition, current studies have merged this ECGi technology with computational models to provide patient-specific models in order to predict the efficacy of specific therapies (Boyle et al., 2018; Huntjens et al., 2018). Due to the influence of inhomogeneities inside the torso on the BSP, recent studies have also focused on the understanding of the forward problem and its relation with the inverse solution (Bear et al., 2015, 2018b; Zemzemi et al., 2015). Furthermore, the ill-posed nature of the problem requires different mathematical constraints and regularization methods to be used to find the most accurate physical and physiological solution (Oster and Rudy, 1992); recent studies have investigated the accuracy of these inverse methods (Bear et al., 2018b).

Despite these important works, there are still many questions in the field of ECGi which must be addressed in order to further develop the approach and improve its clinical and research impact. In this study, we provide analysis of the impact of electro-anatomical variability pertaining to differences in cardiac anatomy and heart rate on the accuracy of ECGi reconstructions obtained using different regularization methods. These analyses provide important insights for the interpretation of clinically obtained ECGi reconstructions over the time-course of an electro-anatomically dynamic condition such as heart failure.

#### Wall Thickness and Heart Size

Previous studies have investigated the influence of tissue inhomogeneities on the BSP and reconstructed solution, which were shown to have a small impact on the reconstructed signal (Ramanathan and Rudy, 2001; Zemzemi et al., 2015). Effects such as an enlargement of the heart and thickening of the cardiac wall (associated with various disease states, e.g., heart failure) are not necessarily included in the geometrical transfer matrix, and have not been fully studied. In this study, we first compared the BSP activation maps obtained by modifying the size of the ventricles. This was used as the baseline comparison between BSP prior to obtaining the inverse solution. Using RMS, rRMSe, and PCC to quantify the similarity or differences between the BSP observed under these different conditions demonstrated that cardiac anatomy had a measurable effect on the details of the BSP but did not significantly alter the primary spatio-temporal features of normal activation (Figure 2).

Then, we observed how modifying the anatomy of the ventricles in the forward solution but not in the inverse approach had an effect on the accuracy of reconstructed ectopic activation. Larger RMS and rRMSe values were observed when comparing BSP error values vs. reconstructed error values (Figure 3; Table 2). Mostly, the first part of the activation (first 75 ms) produced the most significant differences. However, it was still possible to identify the origin of ectopic activation, albeit with a small error (Figure 4A).

These results therefore indicate that it may not be necessary to repeat a cardiac CT/MRI when repeating ECGi in a patient who has undergone anatomical remodeling since their first ECGi procedure, which could significantly reduce the cost of long-term treatment. Some consideration may still be required to determine appropriate electrode positions—especially if the torso, as well as the heart, has undergone anatomical changes.

#### Heart Rate

Heart rate had a regularization approach-dependent effect on the accuracy of reconstructed activation patterns: In the case of the First and Second order the PCCs were larger at slow rates but significantly decreased when the heart rate was increased, exhibiting a negative temporal evolution over the activation period. In the case of Zero order, the values, even though not larger than the First or Second order in any case, remained similar when the heart rate increased and stable over the activation time at rapid rates (Figure 5; Table 3). The ED also showed an important dependency on the heart rate, increasing when the heart rate increased (Figure 4B). Therefore, these data indicate that the accuracy of inverse solutions in general decreases at rapid pacing rates. The underlying cause of this order-dependent difference in the rate-dependence of the solution is discussed in the next section, “On the Rate-Dependence of Time-Independent Solutions.”

Differences in the quality of reconstruction at fast rates may have particular clinical importance: higher rates may present the most clinically interesting results, for example exposing concealed abnormalities (Leong et al., 2018), yet produce the poorest reconstructions using ECGi. This may also indicate that rapid arrhythmias such as tachycardia or fibrillation could present the greatest challenges for reconstruction, additional to the spatial complexity of the excitation pattern itself.

### On the Rate-Dependence of Time-Independent Solutions

We observed that the accuracy of the solution using both First and Second order methods was rate-dependent, resulting in larger errors and smaller correlations at rapid rates; a feature not observed using the Zero order method (Figures 4, 5; Table 3). This rate dependence raises an interesting question: given that the solutions to the inverse problem of electrocardiography use a quasi-static approximation, how does a temporal effect such as pacing rate modify the quality of the solution?

This can be explained by examining the differences between the regularization methods: A primary difference between the Zero order and First and Second order approaches is that the Zero order approach does not include any neighbor interaction (as it uses the identity matrix as the regularization operator) whereas the First and Second order do account for this interaction and result in smoothed signals (due to the use of the Gradient and Laplace operator for First and Second order, respectively). Spatial heterogeneities in voltage will therefore be smoothed using First and Second order but not Zero. Following that this approach distinction correlates with whether or not the reconstruction exhibits rate-dependence, we propose that spatial gradients observed at rapid rates, not present at slower pacing rates, may account for this observation.

In a previous preliminary study in the atria (Alday et al., 2016) we presented the hypothesis that this was primarily due to shortening of the AP morphology at rapid rates, resulting in a short excitation wavelength and therefore the simultaneous presence of both depolarization and repolarization wavefronts from a single excitation, significantly enhancing spatial gradients at temporal snapshots during the activation. An alternative explanation is that it is the presence of regions of tissue still active from the previous excitation at the time of the stimulus which lead to these enhanced spatial gradients. We tested which of these hypotheses was more likely to underlie the observation by comparing the control data at pacing cycle lengths of 300 and 1,000 ms with new simulations at those cycle lengths in which the AP duration has been significantly shortened (Figure 1Biii): this captures the shorter wavelength associated with rapid control pacing while simultaneously imposing that the previous excitation is no longer or only minimally present in the tissue at the time of excitation.

**Figure 6:**
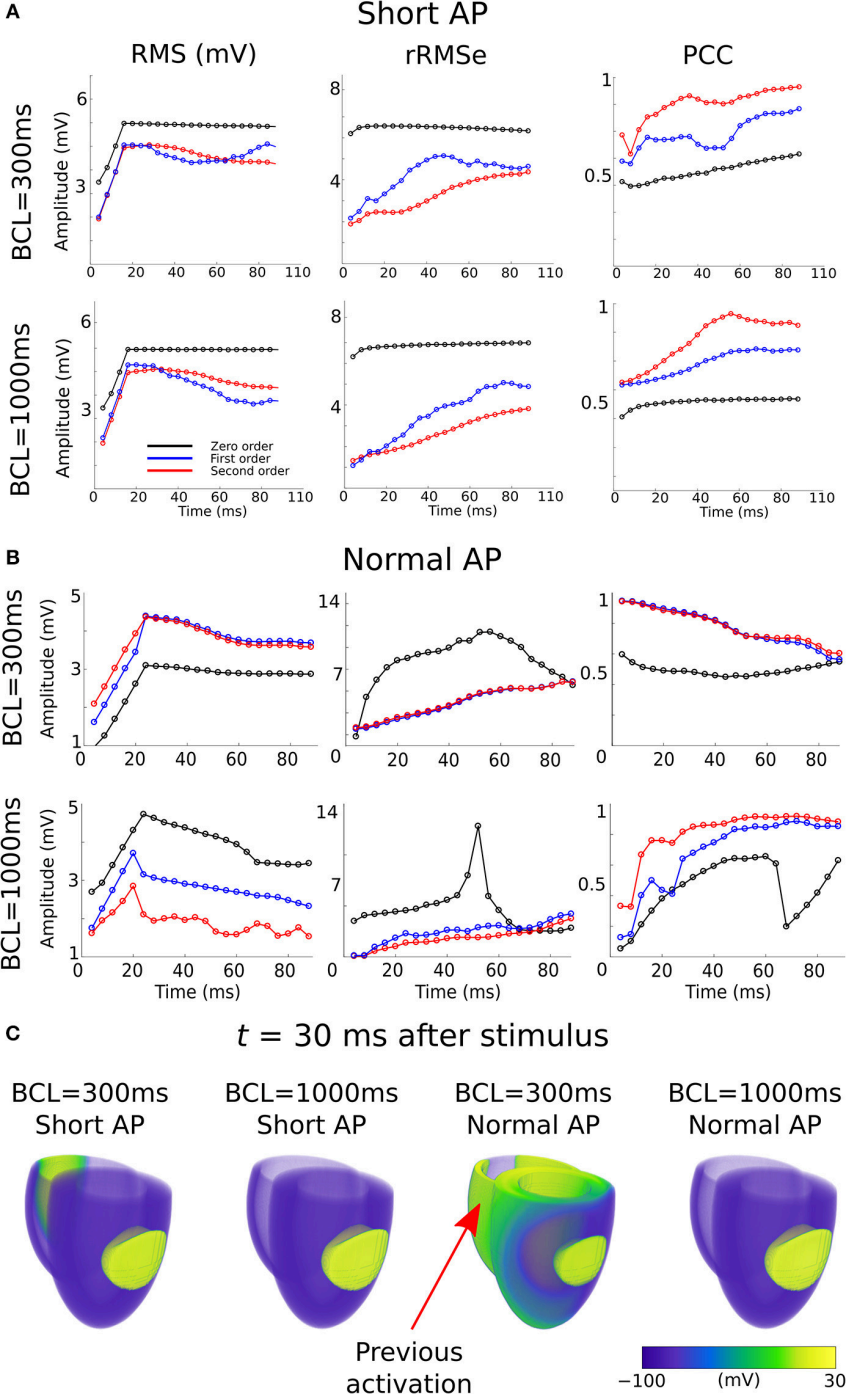
RMS, rRMSe, and PCC of the reconstructed activation maps using **(A)** short and **(B)** normal AP durations at fast (BCL = 300 ms; upper panel) and slow (BCL = 1,000 ms; lower panel) pacing rates, for the Zero (black), First (blue), and Second (red) order Tikhonov regularization methods. **(C)** Corresponding snapshots of propagation at time *t* = 30 ms after stimulus of the LV-LAT ectopic site. The control geometry was used in all cases.

The RMS and PCC of First and Second order reconstructions associated with both the pacing rates using the short AP model were comparable to the slow pacing rate using the control AP model, and differed from the rapid pacing rates (Figure 6; Section S2 in Supplementary Material). In particular, the shorter AP models did not reproduce the negative temporal evolution and general lower PCC observed at the rapid rates using the control AP. These data indicate that, contrary to our original hypothesis, these increased errors were not caused by the short excitation wavelength (where it would be expected that all short AP models reproduced these features) but were rather caused by the presence of the previous excitation; the only condition which reproduced the lower correlation and large errors was the one in which large areas of still active tissue remained at the time of excitation (Figure 6C). The temporal evolution of the PCC also indicates that it is the presence of two large and distinct regions of active tissue, rather that multiple depolarization and repolarization wavefronts, which induces the reconstruction errors: the initial large PCC observed at rapid rates for control, which contrasts with the less accurate estimation of the focal location (i.e., increased ED), is a result of accurate reconstruction of the large area of active tissue from the previous excitation; as the area of active tissue from the present excitation grows, the correlation decreases due to inaccurate reconstruction of two large regions. There is no large impact on the PCC at the time the previous excitation's depolarization wavefront terminates at full activation; rather, the lower correlation remains until the tissue repolarizes.

### Limitations

These types of studies are key to fully translate ECGi technology into clinical settings. However, they are difficult or impossible to perform in control and experimental settings, and the accuracy of the forward and inverse solution is still under study (Bear et al., 2015, 2018b). Computational modeling offers an important tool to study, understand and provide insights into the effects of cardiac arrhythmias and clinical variables (Colman et al., 2013, 2017). Unfortunately, there are still several limitations that need to be addressed. The forward model lacks several inhomogeneities which may have an important effect on the BSP measured and therefore in the reconstructed signal, as we used a homogeneous torso approach for the inverse reconstruction. However, previous studies have shown that the effects of inhomogeneities in the inverse solution are small (Ramanathan and Rudy, 2001);(Zemzemi et al., 2015).

Another limitation is the idealized ventricular models used, which lacked the complex anatomy and microstructure of the real human ventricles (Stephenson et al., 2017). However, these were implemented to facilitate investigation of the effects of changing the size and wall thickness of the ventricles on non-invasive mapping. We used standard approaches to model cellular and ventricular electrophysiology, the general limitations of which have been addressed in detail elsewhere (Benson et al., 2011).

Due to both the BSP and inverse solution being computed using simulations, it is important to ensure that “inverse crime,” where the inverse method exactly inverts the forward method, is avoided. We ensure that this is the case through the use of Salu's method for the forward problem and Barr's for the inverse solution; thus, whereas the forward problem is solved by computing the electric field which arises as a result of currents in the cardiac tissue, the inverse solution uses an equivalent potential distribution on a closed surface. These independent methods, utilizing two different matrices, ensure that it is not possible for the inverse solution to exactly invert the forward solution.

Only ventricular activation was considered for comparison between different rates and geometries, disregarding potential analysis of the repolarization patterns, which may themselves provide substantial diagnostic information. Future investigation of the effects of anatomical reconstruction inaccuracies and heart rate on the reconstruction of repolarization patterns may therefore provide valuable information. However, the present study was focused on identifying the location of ectopic pacing sites, relevant in particular for guiding ablation therapy, and therefore requires only activation patterns to be reconstructed.

There are multiple further factors which will be relevant for clinical studies but not accounted for in the idealized and controlled *in silico* experiments of the present study. Whereas the geometry of the heart was considered, this was not combined with analysis of its location, its mechanical movement, or electrode location errors, all of which have been previously shown to be important factors influencing the accuracy of the reconstruction (Swenson et al., 2011; Cluitmans and Volders, 2017; Cluitmans et al., 2017; Coll-Font and Brooks, 2018). Furthermore, we did not investigate whether the effect of heart rate was influenced by the geometry, treating these analyses as separate; such investigation may provide further important insight.

In addition, the inverse problem of electrocardiography is an ill-posed problem and therefore noisy signals can have an important effect of the reconstructed maps. Whereas, the simulated data in this study and used for our analyses did not include noise, we performed further simulations in which white noise was included. These data demonstrated that noise increased ED and decreased correlation, but the differences between conditions were maintained, indicating that whereas noise has an important impact on the activation maps and ED, our observations about different geometries and pacing rate are maintained (Section S3 in Supplementary Material; Figures S4–S7, and Tables S1, S2). Also, the results obtained in this study are of the same order of magnitude observed in previous studies (Wang et al., 2010; Bear et al., 2018b);(Tate et al., 2018).

## Conclusion

The systematic analysis revealed that the effect of size, thickness, and heart rate can manifest in the BSP and ECGi in different ways, with varying sensitivities and success rates in inferring the clinical variables from non-invasive information. We observed a rate dependence in the ability of different Tikhonov regularization methods to successfully reproduce cardiac electrical activity. Our results show that the ECGi approach gives the most accurate results when used with geometries depicting the current state of the patient's heart, but if a single image of the patient's heart is obtained, for example at the start of treatment, the ECGi approach still gives useful and reasonably accurate information relating to underlying electrophysiological abnormalities. In addition, clinical variables such as heart rate need to be accounted for when solving the inverse solution, in particular due to the increase in errors observed at rapid pacing rates.

## Author Contributions

EP conceived the study. EP and MC designed the study. DW developed, applied, and analyzed data associated with the bi-ventricle cardiac excitation model. EP developed, applied, and analyzed data associated with the forward problem, inverse solution, ECG, and error analysis. EP and all other authors interpreted the data. EP and DW prepared the figures. EP and MC prepared, and all authors edited, the drafts of the manuscript. All authors approve the final version of the manuscript and agreed to be accountable for all aspects of the work in ensuring that questions related to the accuracy and integrity of the work are appropriately investigated and resolved.

### Conflict of Interest Statement

The authors declare that the research was conducted in the absence of any commercial or financial relationships that could be construed as a potential conflict of interest. The reviewer JT declared a past collaboration with one of the authors EP to the handling editor.
