# Supplementary material for: Effects of Heart Rate and Ventricular Wall Thickness on Non-invasive Mapping: An in silico Study
Source: Front Physiol. 2019 Apr 5;10:308. doi: 10.3389/fphys.2019.00308 (PMC6460935; doi:10.3389/fphys.2019.00308)
Supplement: Supplementary file 1 [file Data_Sheet_1.pdf]

Supplementary Material

Effects of heart rate and ventricular wall thickness on non-invasive mapping: an *in silico* study

Erick Andres Perez Alday[1*], Dominic G. Whittaker[2], Alan P. Benson[2], Michael A. Colman[2*]

[1]Knight Cardiovascular Institute, Oregon Health & Science University, Portland, OR, United States of America.

[2]School of Biomedical Science and Multidisciplinary Cardiovascular Research Centre, University of Leeds, Leeds, United Kingdom.

***Correspondence:**
Erick Andres Perez Alday, perezald@ohsu.edu

Michael Alan Colman, m.a.Colman@leeds.ac.uk

Table of Contents

S1 – CARDIAC ACTIVATION MAP CONSTRUCTION 2

S2 – ACTIVATION MAPS UNDER DIFFERENT RATES AND APD 3

S3 – THE EFFECT OF NOISE 3

S3.1 WHITE NOISE 3
S3.2 EFFECTS OF WALL THICKNESS AND HEART SIZE 4
S3.3 EFFECTS OF HEART RATE 5

REFERENCES 7

S1 – Cardiac activation map construction

As described in the manuscript, cardiac activation maps were calculated by computing maximal negative slope at each node at each time step (Gage et al., 2017). This mean that we solved the inverse solution for the whole ventricular activation and then find the maximum negative slope at each node (Figure S1).

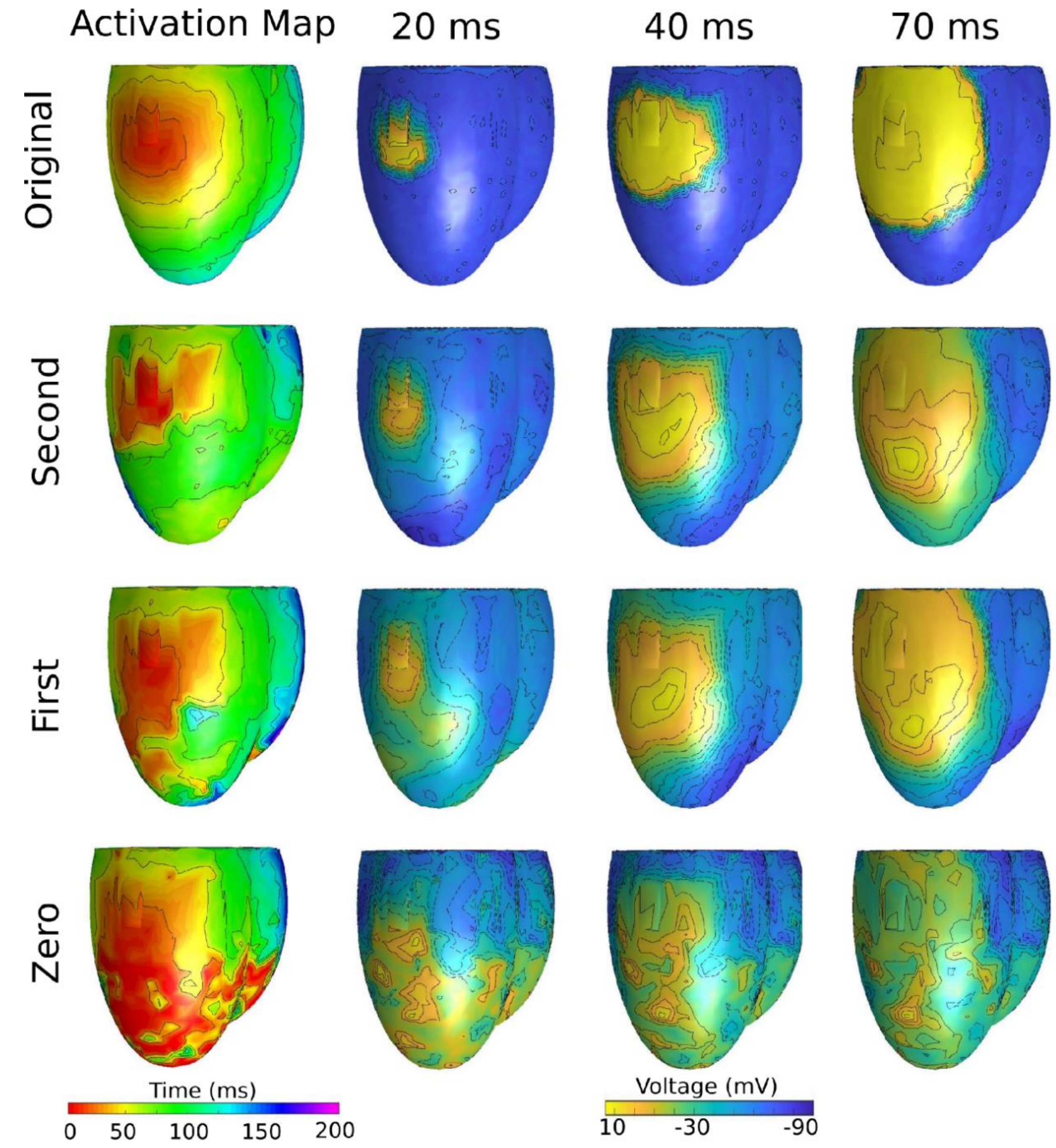

Figure S1: Activation maps and snapshots of epicardial potential reconstruction of an ectopic activation initiated in the right ventricular lateral wall. (A) Activation map and snapshots at (B) 20ms, (C) 40ms, and (D) 70ms of epicardial potentials of an ectopic activation initiated in the right ventricular lateral wall (RV-LAT) on the control geometry. Original activation patterns were compared with the Zero, First and Second order Tikhonov regularization reconstructions.

Activation patterns were computed as the time of maximum first derivative d*V*/*d*t at each location. Normalized amplitude were used to compare the snapshots.

S2 – Activation maps under different rates and APD

Zero, First and Second order Tikhonov regularization were used to compute non-invasive cardiac maps for two different pacing rates but the same short AP duration, for an activation starting on the middle of the left ventricle (LV-LAT; Figure S2). The same ventricular geometry (control) was used in all cases for both forward and inverse problem.

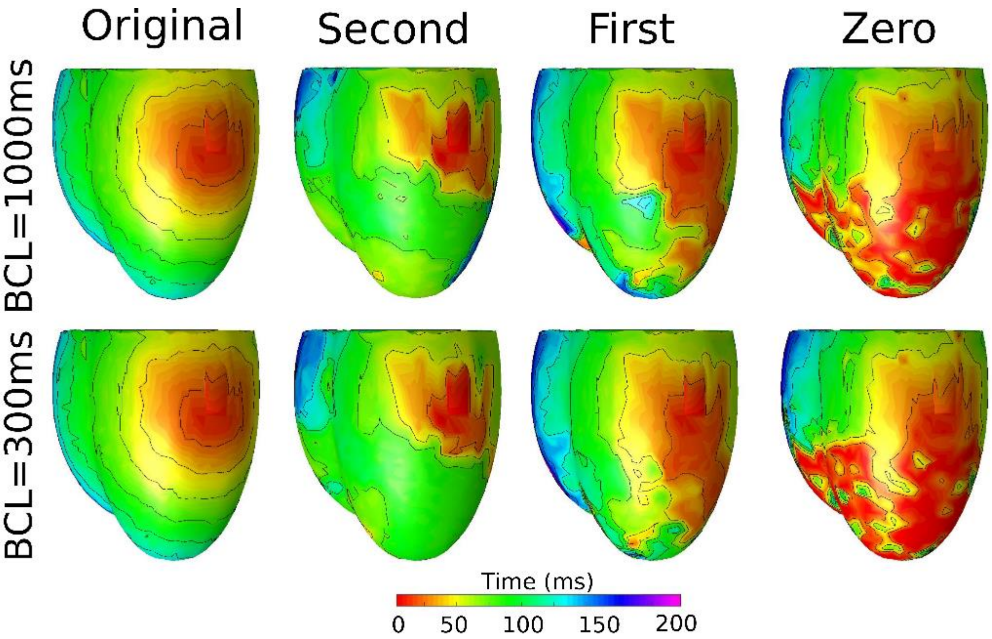

Figure S2. Non-invasive cardiac maps reconstructed using the same short AP duration at a slow (upper panel) and fast (lower panel) pacing rate, using the Zero, First, and Second Tikhonov regularization methods. Activation started from LV-LAT. Control geometry was used in both cases.

S3 – The effect of noise

The inverse problem in electrocardiology is an ill-posed problem, hence the influence of noise can have an important effect on the solution obtained. Therefore, noise was added to the body surface potential (BSP) to test whether the addition of noise would affect any of our primary observations.

S3.1 White noise

White noise was added to the BSP obtained from ectopic activations with origins in (i) the intra-ventricular septum (SEP) and (ii) the left ventricular apex (LV-Apex).

White noise (σ) at 20% was added using, *Rnd,* random numbers, *RMS,* the root mean square value of the BSP, and, *f,* the percentage of noise to be added, i.e. *f*=.2 for 20% noise level, through the formula:

$$\sigma = Rnd * RMS * f$$

A signal to noise ratio (SNR) of 10 was used in all cases (Figure S5).

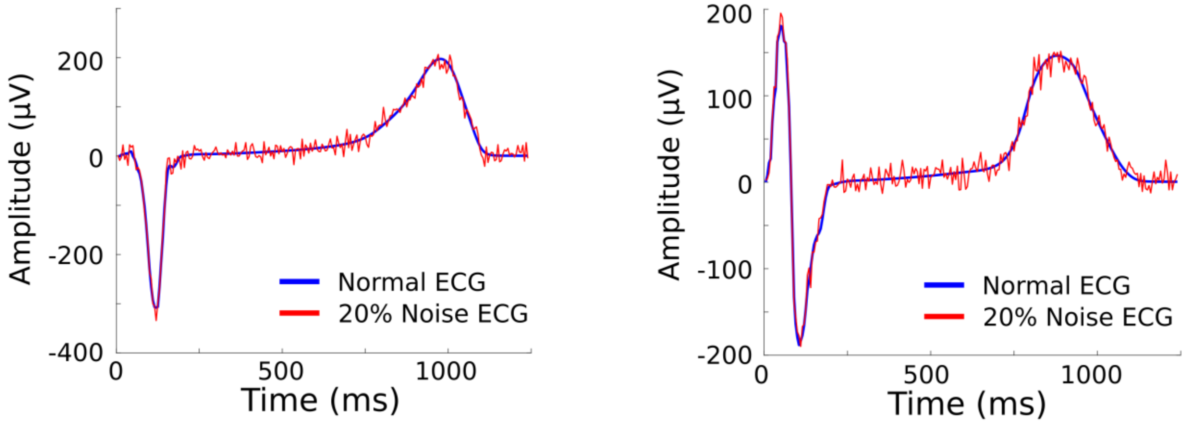

Figure S3. Effects of the white noise added to the normal ECG. Signal from lead V1 and Lead 1.

S3.2 Effects of wall thickness and heart size

The impact of noise on the reconstructions obtained from underlying cardiac anatomy which differs from the geometry employed in the forward method was assessed through analysis of the activation patterns with stimuli applied at SEP focal site in the normal, hypertrophied and dilated geometries. Activation patterns were computed from the time of maximum d*V*/d*t* at each location (Figure S4). All reconstructions were performed on the normal geometry using the Second order Tikhonov regularization methods.

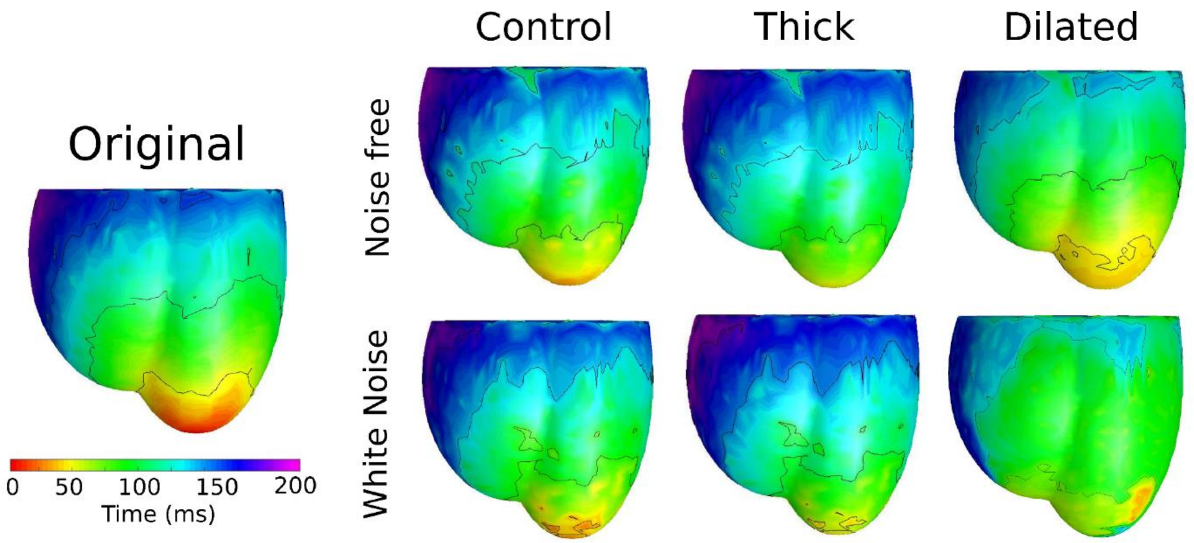

Figure S4. Effects of the white noise on the non-invasive cardiac maps reconstructed for control, thick and dilated geometries. Activation started from LV-Apex. Second order Tikhonov regularization was used in all cases.

Voltage root mean squared (RMS), relative RMS error (rRMSe), and Pearson correlation coefficient (PCC) were calculated at each instant of time to compare the results (Figure S5).

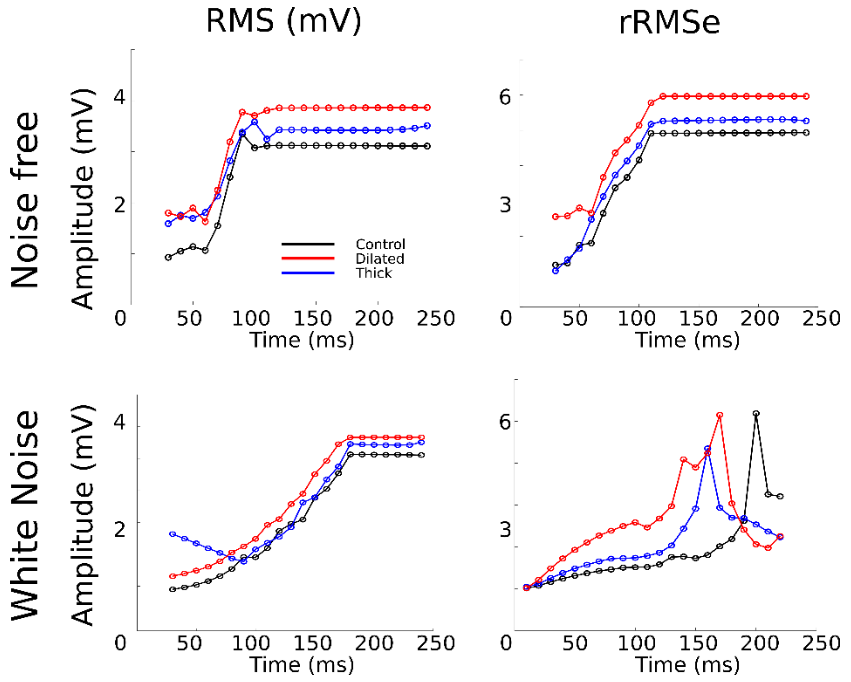

Figure S5. Effects of white noise on RMS, rMSe and PCC calculated for control (black), dilated (red) and thick (blue) geometries. Second order Tikhonov regularization was used for an ectopic activation starting on LV-Apex.

Table S1. Effects of white noise on Euclidean distance (ED) calculated for control, thick and dilated geometries. Activation started from LV-Apex. Second order Tikhonov regularization was used in all cases.

**Condition**	**Control - ED (mm)**	**Thick - ED (mm)**	**Dilated – ED (mm)**
Noise free	8.21	8.65	9.33
White noise	9.78	9.97	10.23

S3.3 Effects of heart rate

The effect of heart rate on the epicardial reconstructions obtained using the second order Tikhonov regularization methods was assessed for focal stimuli applied at LV-Apex, and paced at 60 and 120 BPM (corresponding to basic cycle lengths (BCLs) of 1000 and 500 ms). (see Methods: Ventricular simulation protocols). The activation patterns were computed and compared in Figure S6.

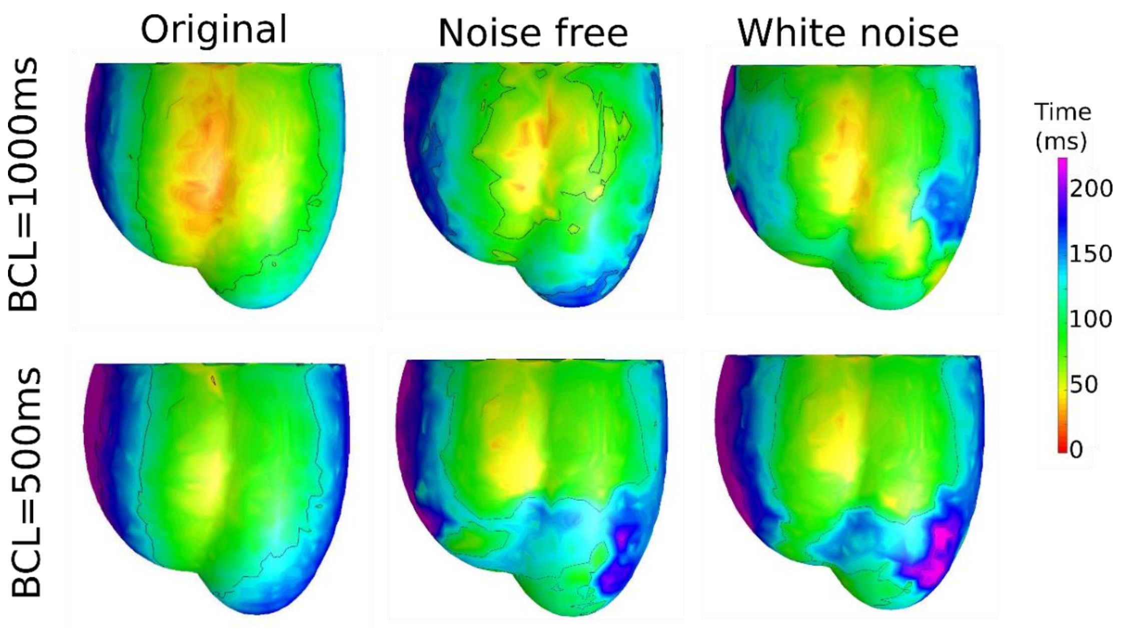

Figure S6. Effects of the white noise on the non-invasive cardiac maps reconstructed for BCL=500 mx and BCL=1000 ms. Activation started from SEP. Second order Tikhonov regularization was used in all cases. Control geometry was used in all cases.

Voltage root mean squared (RMS), relative RMS error (rRMSe), and Pearson correlation coefficient (PCC) were calculated at each instant of time to compare the results (Figure S7).

The white noise added had an important effect on the inverse solution obtained, however, the effects of the geometry and heart rate described in the main manuscript was still observed.

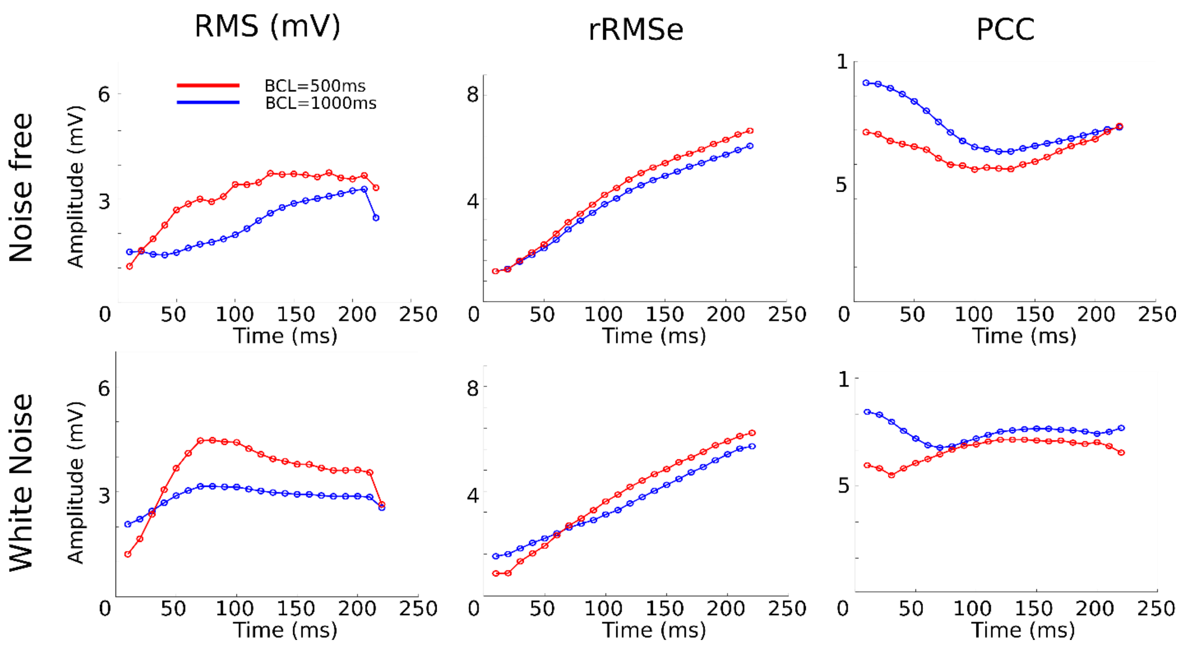

Figure S7. Effects of white noise on RMS, rMSe and PCC calculated for BCL=500 (red) and BCL=1000 ms (blue). The control geometry was used in all cases. Second order Tikhonov regularization was used for an ectopic activation starting on the middle of the left ventricle (LV-LAT).

Table S2. Effects of white noise on Euclidean distance (ED) calculated for BCL = 500 ms and BSL= 1000 ms. Activation started from the middle of the left ventricle (LV-LAT). Second order Tikhonov regularization and control geometry were used in all cases.

**Condition**	**BCL = 500ms – ED (mm)**	**BCL = 1000 ms - ED (mm)**
Noise free	8.74	10.78
White noise	9.94	11.36

References

GAGE, R. M., CURTIN, A. E., BURNS, K. V., GHOSH, S., GILLBERG, J. M. & BANK, A. J. 2017. Changes in electrical dyssynchrony by body surface mapping predict left ventricular remodeling in patients with cardiac resynchronization therapy. *Heart Rhythm,* 14**,** 392-399.
